# Endocrine Effect of Some Mycotoxins on Humans: A Clinical Review of the Ways to Mitigate the Action of Mycotoxins

**DOI:** 10.3390/toxins15090515

**Published:** 2023-08-23

**Authors:** Klaudia Kościelecka, Aleksandra Kuć, Daria Kubik-Machura, Tomasz Męcik-Kronenberg, Jan Włodarek, Lidia Radko

**Affiliations:** 1 Department of Pathomorphology, Faculty of Medical Sciences in Zabrze, 3 Maja St. 13, 41-800 Zabrze, Poland; klaudia.koscielecka@gmail.com (K.K.); aleksandra.kuc@interia.pl (A.K.); daria.kubik64@gmail.com (D.K.-M.); 2 Department of Preclinical Sciences and Infectious Diseases, Faculty of Veterinary Medicine and Animal Sciences, Poznan University of Life Sciences, Wolynska St. 35, 60-637 Poznan, Poland; jan.wlodarek@up.poznan.pl

**Keywords:** mycotoxins, aflatoxin, zearalenone, human, reproductive interference, risk assessment, strategies reduction exposure

## Abstract

Fungi such as *Aspergillus* spp. and *Fusarium* spp., which are commonly found in the environment, pose a serious global health problem. This study aims to present the results of epidemiological studies, including clinical cases, on the relationship between human exposure to some mycotoxins, especially zearalenone and aflatoxin, and the occurrence of reproductive disorders. In addition, examples of methods to reduce human exposure to mycotoxins are presented. In March 2023, various databases (PubMed, Google Scholar, EMBASE and Web of Science) were systematically searched using Google Chrome to identify studies evaluating the association between exposure to mycotoxins and the occurrence of complications related to impaired fertility or cancer incidence. The analysed data indicate that exposure to the evaluated mycotoxins is widespread and correlates strongly with precocious puberty, reduced fertility and increased cancer incidence in women and men worldwide. There is evidence to suggest that exposure to the Aspergillus mycotoxin aflatoxin (AF) during pregnancy can impair intrauterine foetal growth, promote neonatal jaundice and cause perinatal death and preterm birth. In contrast, exposure to the Fusarium mycotoxin zearalenone (ZEA) leads to precocious sexual development, infertility, the development of malformations and the development of breast cancer. Unfortunately, the development of methods (biological, chemical or physical) to completely eliminate exposure to mycotoxins has limited practical application. The threat to human health from mycotoxins is real and further research is needed to improve our knowledge and specific public health interventions.

## 1. Introduction

The endocrine system is a complex network of chemicals that interact with each other, as well as with other systems. It is responsible for the proper functioning of the body and maintaining homeostasis. Dysfunction of the endocrine system leads to severe diseases and ailments that can significantly impede function. There are many causes of endocrine dysfunction. One factor is the common but often ignored and overlooked endocrine disrupting chemicals known as endocrine disruptors (EDCs). According to the World Health Organization (WHO) definition, an endocrine disruptor is “a substance that alters one or more functions of the endocrine system and consequently causes adverse effects in an intact organism, its offspring or (sub)population” [1]. These substances interfere with the system at various stages, such as biosynthesis, transport, metabolism, hormone action at the receptor and post-receptor level or feedback, but they can also interact at the genome level, affecting gene expression and genomic imprinting [2]. This article focuses on a broader presentation of the subgroup of EDCs that are mycotoxins, mainly zearalenone (ZEA) and aflatoxins (AF) but also other mycotoxins that have been linked to endocrine disorders in various studies, i.e., deoxynivalenol (DON), T-2 toxin and fumonisins. Mycotoxins, depending on where they are produced, are classified into two major groups: field-derived and stored. The first group (field-originated) includes mycotoxins from fungi of the genus *Fusarium* spp. (deoxynivalenol, zearalenone, T2-toxin, fumonisins). The second group (storage) includes mycotoxins from *Penicillium* spp. (patulin, ochratoxin and others) and *Aspergillus* spp. (aflatoxin, ochratoxin and others). It is estimated that mycotoxin contamination of food may involve as much as 25% of the world’s agricultural industry products, which is a global problem [3]. The most commonly studied and analyzed EDs-affected mycotoxin is zearalenone. It is a non-steroidal, oestrogenic mycotoxin that is a metabolite of fungi of the genus Fusarium: *Fusarium culmorum*, *Fusarium equiseti*, *Fusarium graminearum*, *Fusarium cerealis*, *Fusarium semitectum* and *Fusarium crookwellensen* [4]. After entering the body, zearalenone is absorbed very quickly; in the plasma of pigs, it was detected as early as 30 min after feeding [5,6]. The main metabolites of zearalenone are α-zearalenol (α-ZEA) and β-zearalenol (β-ZEA), both of which are formed by the reduction of ZEA in the intestinal cells [7,8]. Zearalenone exists in two forms: cis and trans, with the former form having a higher affinity for oestrogen receptors. Both zearalenone and its metabolites can affect the reproductive system, as they are similar in structure to 17β-estradiol, which is an endogenous oestrogen [9]. The study found that ZEA, α-ZEA and β-ZEA can disrupt endocrine metabolism, as they stimulate the production of estradiol, progesterone, testosterone and cortisol in the H295R cell line derived from the human adrenal gland [10]. Sources of ZEA in human life can be food products from mycotoxin-exposed animals (milk, meat or eggs) or other contaminated foods, such as cereals, wine, beer, spices and dried fruits; contaminated water or even air (inhalation exposure) are additional sources. For this reason, organisations such as the European Commission and the European Food Safety Authority (ESFA) have set a daily exposure limit for ZEA [11,12]. Zearalenone is metabolised mainly by enterocytes and hepatocytes, and its conjugated metabolites are excreted in the bile, as well as through intestinal and hepatic circulation and are eventually eliminated in the urine [13]. Elimination is relatively slow [6], which promotes the accumulation of ZEA in tissues [5]. Among mycotoxins, we also distinguish aflatoxins, with aflatoxin B1 (AFB1) representing the group. AF are secondary metabolites of molds, mainly *Aspergillus flavus* and *Aspergillus parasiticus* [14]. AFB1 enters the body through the oral route and is absorbed in the small intestine. Aflatoxin B1 itself is not toxic to humans, but its metabolites, which are formed as a result of AFB1 activation by liver microsomes AFB1-exo-8, 9-epoxide (AFBO) and AFB1 hydroxylation-forming AFM1, are toxic. Their cytotoxic adducts can be responsible for mutations in DNA and lead to acute and chronic diseases [15,16].

In recent years, advances in the fungal genome and transcriptome sequencing, computational tools, gene disruption techniques and analytical chemistry have enabled an understanding of several molecular aspects of mycotoxin biosynthesis and their regulation [17]. A biosynthetic gene cluster (BGC) enables coordinated regulation of their expression. Synthases or key synthetase genes are present in the cluster, along with additional enzymes that modify and form the final complex molecular structure of the mycotoxins and possibly allow transport or reduce their toxicity. Recent studies have demonstrated the critical role of signaling molecules and pathways in controlling fungal responses to many nutritional, chemical and environmental stimuli that affect basic mycotoxin metabolism and biosynthesis [18]. 

AF are the most widely studied mycotoxins in terms of biosynthesis and molecular regulation. Over 19 identified AF analogues are produced by over 16 *Aspergillus* spp. A biosynthetic gene cluster of approximately 30 genes has been characterised in both the major aflatoxin species: *A. flavus* and *A. parasiticus*, although molecular studies have mainly been conducted on the production of AFB1 by *A. flavus* [19]. 

Because of the effects that AFB1 metabolites cause in the body, this substance has been hailed as the most harmful, toxic and carcinogenic aflatoxin. The main reservoir of AFB1 is cereals. Exposure also occurs from the consumption of corn or peanuts. The amount of their consumption proportionally correlates with detected toxin concentrations in biological samples [20]. 

## 2. Methodology

In March 2023, an extensive manual search of major electronic databases (PubMed, Google Scholar, EMBASE and Web of Science) was conducted to identify relevant articles published on the impact of mycotoxins on human health, with a particular focus on the reproductive aspect. No articles published after that date were specified. The articles were limited to those published in English and Polish. Many search terms were used, including “mycotoxins”, “mycotoxins and human health”, “mycotoxins and cancer”, “mycotoxins and reproduction”, “zearalenone” and “aflatoxins”. The keywords used to obtain data on the reproductive effects of mycotoxins, especially aflatoxins and zearalenone on human reproduction, included “myco-toxins*human health”, “aflatoxins*human health”, “zearalenone*human health”, “mycotox-ins*cancer”, “aflatoxins*cancer”, “zearalenone*cancer”, “mycotoxins*human reproduction”, “aflatoxins*human reproduction”, “zearalenone*human reproduction”, “mycotox-ins*detoxification”, “aflatoxins*detoxification”, “zearalenone*detoxification” and “mycotoxins*children”. The articles were analyzed first by title, then by abstract, and finally by the full text. All the selected articles were the most relevant ones available for this review. The data from the timeframe of 1981–2023 were used to search for information on mycotoxins and reproduction, and data from 1997–2022 were used to search for information on detoxification. A total of 124 articles were found.

### 2.1. Mycotoxins and the Human Reproductive System

Exposure to mycotoxins present in the environment can impact the entire life of an individual from the foetal period to adulthood, affecting most body systems. An altered hormonal balance caused by EDCs, which include mycotoxins, is responsible for this [21,22]. EDCs negatively affect, among other things, the function of growth-promoting hormones, such as insulin or insulin growth factors 1 and 2; in parallel, they also promote the action of glucocorticoids, which in turn inhibit this process [23]. ZEA as EDCs may also affect endocrine function of the placenta and kidneys, which is associated with selective inhibition of HSD11B2 [24]. According to other authors, such a phenomenon may be caused by pro-inflammatory effects of ZEA or with receptors for oestrogen [25]. Other examples of hormones that do not function properly as a result of interference with EDCs are growth hormones produced by the pituitary gland or thyroid hormones. Their excessive or insufficient concentrations have far-reaching implications in individual development, such as the aforementioned growth disorders or delayed puberty in the case of the latter [26,27].

In addition to the many serious consequences of exposure to mycotoxins, it is worth reviewing the topic of sexual development and the reproductive process in the context of exposure to these harmful agents. This topic is important because it may determine the further development of the population. Epidemiological studies in humans show that ZEA has a significant impact on the onset of precocious sexual maturation in girls with simultaneous high concentrations of oestrogen [28,29,30,31]. Similar conclusions were reached by Yum et al., indicating premature pubertal development in boys before age nine [27]. Increased ZEA levels (18.9–103 µg/L in sera) in girls with early thelarche and precocious puberty were also proven in studies conducted in Turkey, Hungary and Italy [28,32,33].

The study by Bandera et al., on the other hand, indicates shortened development and faster breast development in girls aged 9–10 years old [30]. In addition, Rivera-Núñez showed that girls with quantifiable levels of myco-oestrogen were significantly lower than their peers, in whom myco-oestrogen was not observed [31]. ZEA also negatively affects reproduction in both sexes by controlling the production and secretion of such sex hormones as the aforementioned estradiol, progesterone and testosterone, among others, potentially causing infertility [34]. 

Despite the limited number of studies available, scientists favor the thesis that myco-oestrogens are responsible for the significant disruption of ovarian folliculogenesis [35,36]. Mycotoxins may also be complicit in the manifestation of conditions such as polycystic ovary syndrome (PCOS), premature ovarian function (POF) and endometriosis [37], which can cause serious problems in conception and maintaining a pregnancy. ZEA also hinders fertility by affecting male reproductive cells [38]. By binding to oestrogen receptors, it causes an increase in estradiol, which leads to inhibition of luteinizing hormone (LH) synthesis in the pituitary gland. Reduced LH levels lead to a decrease in testosterone levels in Leydig cells [38]. Such altered testosterone reduces the efficiency of spermatogenesis. Reduced sperm motility, viability or acrosomal response also result from the ability of ZEA to bind to receptors on male reproductive cells. The formation of reactive oxygen species on cells that are exposed to ZEA also significantly impairs spermatogenesis [38]. Another mycotoxin suspected of affecting male infertility is AFB1 [39,40]. In a Nigerian study, it was discovered in the semen of infertile men at higher concentrations (60 ng/mL to 148 ng/mL) in contrast to semen samples from fertile men (0 ng/mL to 5 ng/mL) [39]. The same study also showed that sperm parameters, such as motility and reduced count, were more common in men with higher AFB1 levels. A similar study was conducted by Uriah et al. on fertile and infertile men, and they showed that higher concentrations of AFB1 in men with fertility disorders significantly exceeded the standards for this substance established by the WHO [40].

The analyzed data indicate that exposure to mycotoxins, in particular, ZEA and AFB1, is widespread and highly correlates with disorders of sexual development and decreased fertility worldwide. This problem should be more widely understood through new research. It is also very important to raise awareness of what the consequences of exposure to such harmful substances entails.

### 2.2. Mycotoxins and the Placenta

Investigating the direct effects of ZEA on placental functions, such as maintenance of the placental barrier, cell fusion and cell secretion, is a priority [41]. Some authors have reported that stimulated cell fusion, which is a response to ZEA exposure, caused an increase in the secretion of human chorionic gonadotropin (hCG) [42,43]. It is also worth mentioning that α-zearalanol may increase the risk of preterm labor by increasing the production of corticotropin (CRH) and COX-2 [41]. Partanen et al. analyzed AFB1 and its metabolites, demonstrating their content in umbilical cord blood after maternal exposure [44]. Eight human placentas were studied, proving the transfer of AFB1 and its metabolism to aflatoxicol (AFL). The authors also noted the degree of exposure depending on the region of the world [44]. In European Union countries, due to effective control of food products, the degree of exposure is low, which cannot be said of China [44]. Wart et al., on the other hand, demonstrated phase I and II metabolism of ZEA to highly oestrogenic α-zearalenol and ZEA-14-sulfate, as well as rapid transfer of ZEA across the human placental barrier in an ex vivo human placental perfusion model [45]. In conclusion, the unborn child may be exposed to various substances and their metabolites, e.g., ZEA and AF, which may affect hormonal balance.

### 2.3. Mycotoxins and Birth Weight

Mycotoxins are one of the factors affecting the birth weight of a newborn. In the literature, there are many studies in humans that examined the correlation between maternal exposure to mycotoxins and birth weight [46,47,48,49,50,51]. 

Most of them confirmed a negative correlation between birth weight and AF [47,48,49,50].

Turner et al. demonstrated that exposure to AF during pregnancy results in the formation of its metabolite in umbilical cord blood, which is associated with the expression of CYP3A enzymes already present in the uterus [50]. It follows that maternal exposure to AF can adversely affect neonatal weight [50]. Similar conclusions were reached by Shuaib et al. based on a prospective cohort study. In addition, they indicated a reduction in the head circumference of newborns [51]. One older study found no association between the presence of an AF compound in the mother and the birth weight of 625 infants [46].

### 2.4. Mycotoxins and Neonatal Jaundice

The development of neonatal jaundice may be related to maternal exposure to AF [47,52,53].

In 1998, Abulu et al. examined aflatoxins present in cord blood samples from 14 neonates without jaundice and 150 neonates with jaundice [47]. Newborns in the latter group showed high mean AFB1 concentrations (32.3 ng/mL and 35.6 ng/mL) ranging from 5.0 to 30.2 pg/mg. The authors pointed to maternal exposure to mycotoxins during pregnancy [47]. Similar conclusions were drawn by Sodeinde et al., who showed a correlation between serum AF levels in newborns and bilirubin levels and jaundice [52]. On the other hand, other authors have not found the described relationship [49,53].

Given the various research results and concepts presented, it is worth noting the need for further research in this area.

### 2.5. Mycotoxins and Miscarriages and Stillbirths

Studies on the effect of aflatoxin exposure on miscarriage are still lacking. Shuaib et al. showed a 35% higher probability of stillbirths, taking into account the highest maternal exposure to AF [54]. Three major Norwegian studies have analysed the exposure of female farmers to mycotoxins present in grain farming, among other things [55,56,57]. A higher number of premature births (21–24 hbd) and late miscarriages were proven; however, no link was obtained between the toxins and perinatal deaths [55,56,57]. Another study was based on a retrospective interview with 513 postpartum women regarding their diet during the first two months of pregnancy. A positive correlation was obtained between the consumption of foods with aflatoxins and neonatal deaths [58].

### 2.6. Mycotoxins and Birth Defects 

Mycotoxins such as AF, ZEA, ochratoxins and fumonisins can adversely affect the foetus when they cross the placental barrier, leading to malformations of the central nervous system and damaging the brain [59,60]. One Norwegian study investigated cryptorchidism, hypospadias and genitourinary birth defects in males as a result of exposure to mycotoxin. These defects occurred at a higher frequency in children of farmers exposed to fungal metabolites during conception compared to a group of children whose parents were not exposed to mycotoxins [56]. In contrast, Missmer et al. made an estimation of fumonisin levels in maternal blood by means of the sphinganine/sphingosine ratio, and established an association of this ratio with the risk of neural tube defects, such as spina bifida. Above a value of 0.35 of the ratio tested, foetal death was more likely to occur [60].

### 2.7. Mycotoxins and Preterm Birth

According to some authors, aflatoxins present in food ingested during pregnancy can lead to preterm delivery [51,61]. Exposure to AF results in an increase in maternal pro-inflammatory cytokines and, consequently, also in the foetus. This pathological process initiates cervical ripening, rupture of the amniotic membranes and preterm contractions (<30 hbd) [61,62]. Other studies have assessed the effect of AF in the blood on preterm birth and found a high probability [48,61]. Similar conclusions were drawn by Wang et al., proving that zeranol applied at a dose of 1–100 mg/kg/week in mice correlated with early delivery [63]. In contrast, Andrews-Trevino et al. found that due to AF exposure, 13% of newborns from a group of 1621 mothers were born prematurely, indicating a negligible association [64].

### 2.8. Mycotoxins and Anaemia

Many studies confirm the link between aflatoxin exposure and anaemia [51,54,61]. Andretta et al. indicated a significant reduction in haemoglobin and haematocrit as a result of mycotoxins [65]. Other studies link AFB1 to an inhibitory effect on haematopoiesis, erythrocyte haemolysis, reduced and impaired iron absorption, initiation of microcytic anaemia and effects on iron levels [54,66]. In contrast, Smith et al. linked the toxins in question to reduced erythropoiesis, reduced intestinal iron absorption capacity and immune activation and enteropathy [61]. A strong correlation between anaemia (haemoglobin levels < 11 g/L) in pregnancy and aflatoxins (‘low’: < or =2.67, ‘moderate’: >2.67 to < or =4.97, ‘high’: >4.97 to < or =11.34, ‘very high’: >11.34) was identified by Shuaib et al. [54]. The occurrence of microcytic anaemia brought on by AFB1-contaminated food was evidenced in another study involving white rabbits [67]. On the other hand, Smith et al. concluded that the findings presented by the researchers above may not be relevant to humans, given the high doses used on animals [61].

### 2.9. Mycotoxins and Pre-Eclampsia

It is thought that mycotoxins (fumonisins) may be one of the causes of pre-eclampsia. One of them, fumonisin B1, can induce the generalised inflammatory response associated with pre-eclampsia. One study revealed that the lowest mean blood fumonisins B levels were in normotensive women, higher in pre-eclamptic pregnant women and highest in eclamptic pregnancies [68]. In contrast, in another study using surrogate measures for mycotoxin exposure, no correlation was found between pre-eclampsia and cereal farming, but it was noted for animal farming [57]. There was no association between exposure to crop-related inhalation endotoxins and pre-eclampsia (aRR = 0.93; 95% CI 0.86–1.01), but there was an association between animal breeding and pre-eclampsia (aRR = 1.14; 95% CI 1.07–1.22) [57]. No studies showing an effect of ZEA or AFB1 levels on the presence of pre-eclampsia were identified.

### 2.10. Mycotoxins and Cancer

Various studies have examined the links between the process of carcinogenesis and naturally occurring oestrogen disruptors [4,56,59,69,70,71,72,73,74,75,76,77,78,79,80]. The link between exposure to xeno-oestrogens in early development and the occurrence of chronic diseases, such as cancer in later life, also appears to be important [70,71]. It is likely that zearalenone, as one of the representatives of mycotoxins, through its effects on gonadal and pituitary function can disrupt the endocrine system in humans. Some authors highlight its role, for example, in the development and progression of breast cancer [69,70,71]. Other researchers indicate that its highly oestrogenic properties may be linked to cancers such as ovarian cancer, cervical cancer, breast cancer and prostate cancer, and that long-term consumption of foods contaminated with this substance may create adverse health effects [72]. In the article by Kuciel-Lisieska et al., a significant percentage (37%) of women with breast cancer were found to have ZEA in their blood; in addition, higher concentrations of this substance of 10.40 ng/mL were reported in patients with benign breast tumours [73]. The researchers concluded that ZEA may be one of the risk factors for this cancer in patients [73]. Additionally, another study assessed the risk of breast cancer from exposure to ZAE and five of its metabolites (α-zearalenol, β-zearalenol, α-zearalanol, β-zearalanol and zearalanone) by evaluating the urinary concentrations of these substances in the women studied, and suggests that α-zearalanol may play a potential role in the risk of developing breast cancer [74]; however, another study found no significant differences in the plasma concentrations of ZEA and its metabolites, α-zearalenol and β-zearalenol, in breast cancer and cervical cancer patients compared to a group of patients with other diagnoses and healthy female volunteers [75]. The researchers report that the crux of the reason for the inconsistency in the studies above may be due to differences in how these mycotoxins are measured [76]. In contrast, the study by Pajewska et al. analysed 12 samples containing endometrial hyperplasia and 49 containing endometrial cancer—a total of 61 samples [77]. It was concluded that cancer cell proliferation in the uterus can be caused by both metabolites and ZEA itself and that these compounds can be accumulated in uterine tissues [77]. An important role in carcinogenesis can also be attributed to AFB1. CYP450 is involved in the metabolism of this compound, and the mutagenic exo-8,9 epoxide forming the 8,9-dihydro-8-(N7-guanyl)-9-hydroxy-AFB1 adduct (AFB1-N7-Gua) becomes crucial [78]. Studies have suggested the involvement of mutations in various genes, such as p53, c-KRAS and HRAS, in AFB1-related tumourigenesis. Prostaglandin H synthase (PGH) may also be involved in epoxidation [78]. In another mechanism, lipid peroxidase (LPO) may be involved, which may inhibit DNA repair by producing an α-methyl-γ-hydroxy-1,N2-propane-dG (met-OH-PdG) adduct [78]. The role of AFB1 in inducing oxidative stress that can damage DNA has also been highlighted. This mycotoxin has been linked to cancers such as hepatocellular carcinoma (HCC), lung cancer, gastrointestinal malignancies, kidney cancer, breast cancer and gallbladder cancer [78]. The researchers pointed out that EDCs (which include mycotoxins) with oestrogenic effects can activate endometrial receptors, promoting proliferation and even neoplastic transformation of hormone-sensitive tissues. They emphasise the importance of lifestyle in cancer prevention [79]. A link was also found between exposure to mycotoxins (aflatoxins, fumoinsins) and various other adverse health effects, including modification of immune function, impaired growth in children, neural tube defects, oesophageal cancer, liver cancer and death in cases of acute exposure [80].

### 2.11. Exposure, Prevention and Detoxification

Human exposure to contaminants present in food (including mycotoxins) can be carried out through human biomonitoring (HBM) [81]. One method of measuring concentration of mycotoxins is liquid chromatography coupled to a mass spectrometer, which allows the level of myco-oestrogens to be measured, e.g., in urine, allowing human exposure to these substances to be assessed [81]. The presence of ZEA has been detected in populations living in countries located on different continents: Europe (Italy, Belgium, Germany, Sweden, Portugal), Asia (Bangladesh), Africa (South Africa, Cameroon, Nigeria) and North America (Haiti, USA (in Bexar County, Texas) [30,82,83]. Over the years, researchers have studied the concentration of mycotoxins in the urine of patients exposed to a certain dose of mycotoxins. In their study, Mirocha et al. determined the concentration of ZEA and its metabolites in men who had received 100 mg of ZEA after ingestion [84]. The ZEA levels were evaluated at 6, 12 and 24 h after administration of the substance. After 6 h, the presence of ZEA (3.71 μg/mL) and α-ZEA (2.97 μg/mL) was detected without the presence of β-ZEA. After 12 h, all three substances were present and the levels of ZEA (6.87 μg/mL) and α-ZEA (6.00 μg/mL) were higher than after 6 h, while the concentration of β-ZEA was 2.66 μg/mL. After 24 h, levels of all three metabolites were lower than after 12 h (ZEA—2.69 μg/mL, α-ZEA—4.02 μg/mL, β-ZEA—1.97 μg/mL) [84]. Many years later, a similar study was carried out, however, involving one volunteer consuming naturally contaminated food containing 10 μg ZEA and 138 μg deoxynivalenol for 4 days [85]. The researchers examined his urine samples, concluding that ZEA was excreted mainly in the form of glucuronide, and in some samples ZEA-14-glucuronide was determined 3–10 h after exposure. It was also determined that ZEA was excreted at an average rate of 9.4% [85]. Because of the adverse health effects that long-term exposure to mycotoxins can cause, it is important to identify methods of prevention and detoxification of these compounds. According to scientists, to prevent human exposure to mycotoxins, it may be important to reduce plant infections by insects harbouring the fungi, *Fusarium* spp., which produce these toxic substances [2]. Another option seems to be the use of fungicides; however, studies have shown that such treatment can have the opposite effect, as it promotes the production of another mycotoxin—deoxynivalenol (possibly due to an increase in Fusarium infection). The use of fungi and bacteria that reduce mycotoxin levels through the production of antifungal substances was suggested as a beneficial alternative [86]. The role of antioxidants has also been mentioned, but in this case, a major drawback of them is their ease of degradation [86]. The next step is to find methods to detoxify mycotoxins present in human food. It is important to analyse methods to eliminate or at least reduce the toxic effects of ZEA. There are various forms of zearalanone detoxification—chemical, biological and physical [2]. It is possible to use sorbents such as cholestyramine, magnesium trisilicate or aluminosilicate. Reducing the amount of ZEA without removing its metabolites has also been described using yeast and bacteria for this purpose. The use of temperature, irradiation, high concentrations of ozone (O_3_) or H_2_O_2_ can also partially inactivate ZEA [87].

Among the developed methods of detoxifying toxins, biological methods are considered safe. Probiotics such as *Lactobacillus* spp., *Bifidobacterium* spp., the yeast *Saccharomyces cerevisiae* and some *Bacillus* spp. have properties that are useful in removing mycotoxins [88]. Probiotics, in particular, lactic acid bacteria (LAB) (*Lactobacillus rhamnosus*, *Lactobacillus amylovorus*, *Lactobacillus plantarum*, *Lactobacillus pentosus*) and yeasts such as *Saccharomyces* spp., remove mycotoxins through two mechanisms: surface adsorption or biodegradation. Surface adsorption is a fast and reversible process that does not cause chemical changes in the mycotoxin. The results of human clinical trials have shown that the binding capacity of probiotics is one of the best methods of detoxifying toxins [89]. They show great potential for food application, considering their qualified presumption of safety (QPS) status [90]. The second mechanism of action of probiotics is biodegradation, which is a permanent process and can lead to the formation of undesirable (toxic) metabolites [89,91]. Streptomyces strains have been shown to be able to break down mycotoxins AFB1 and ZEA [92]. To implement biodegradation, it is important to monitor the potentially hazardous metabolites and biological effects of the process. Biodegradation, which is able to convert mycotoxins into non-toxic metabolites, has become an alternative strategy for food and feed safety control. Unfortunately, complete detoxification is not possible with single-species strains; therefore, consortia of several/over a dozen microorganisms are used for this purpose, which have been gaining more and more popularity in recent years [92]. In practice, consortia of microorganisms were formed that caused the degradation of single mycotoxins, i.e., AFB1 and ZEA, and the simultaneous degradation of these toxins [93,94,95]. The new solution is the combination of probiotics with enzymes that break down mycotoxins. The development of genetic engineering technology favors the creation of recombinant enzymes that break down single mycotoxins and multi-toxins. An example is research on recombinant peroxiredoxin (Prx) from *Acinetobacter* spp., which degraded about 90% of ZEA in maize [96]. Interestingly, there are some studies that have used the expression of cytochrome P450 from turkey liver to neutralise AFB1 [97]. The commercial application of biological detoxification (biodegradation) technologies requires a lot of research on the health effects of the degradation products in order to develop methods for analysing the resulting metabolites, assessing their toxicity and explaining the mechanisms of degradation. 

Biological methods of combating mycotoxins also include fungi that colonise and live within plant tissues asymptomatically for at least for a part of their life cycle, without causing any harm to the host plants. The application of non-toxic strains of *A. parasiticus* and *A. flavus* has produced exceptional results in the elimination of aflatoxins. Other fungi, such as *Rhizopus* spp., *Trichoderma* spp., *Clonostachys* spp. and *Penicillium* spp., have been successfully used for mycotoxin biocontrol [98]. High hopes are attached to *Trichoderma* spp.—a fast-growing fungus that can parasitise other phytopathogenic and mycotoxin-producing fungi. *Trichoderma* spp. produce a wide range of antibiotic substances [99,100]. Active substances produced by Trichoderma, such as harzianic acid, have been shown to reduce Aspergillus growth and AF production, or possibly inhibit their synthesis [99,100]. The inhibition of different fungal species by *T. harzianum* is comparable and more sustainable and may even be more effective than chemical fungicides. Trichoderma is an active ingredient in commercially available biopesticides, biofertilisers, growth enhancers and natural immune enhancers [100]. Several recent studies have revealed that the use of bioagents and natural products may inhibit the production of AFB1 through the downregulation of its biosynthesis genes, although the molecular mechanism of this process has not yet been understood [88]. 

The use of ultraviolet (UV) radiation has proven to be an effective physical method for reducing pollutants through photochemical degradation and DNA damage, respectively [101]. Aflatoxins are photosensitive and can be degraded by UV exposure [102]. The studies indicate that the irradiation process applied for ZEA detoxification in food can be a safe method [103,104]. UV treatment has been used to degrade mycotoxins in food products. Unfortunately, it has shown many limitations in food applications, such as oxidation of valuable nutrients and low penetration in solids and cloudy liquids [105]. Nevertheless, the method shows promise for practical application in the food industry after assessing the adverse effects on food in terms of sensory and nutritional profiles and toxin residues [105]. 

Magnetic nanoparticles, such as iron and zinc oxides, silver, copper or selenium nanoparticles, are gaining massive attention in effective binding of mycotoxin in feedstuff and foods [106,107,108]. AFB1 degradation has been studied using iron oxide nanoparticles *in vitro* and in edible oils–magnetic graphene composites [109]. The main issue for the practical use magnetic nanoparticles is the lack of the assessment of toxicity and safety limits.

The next method of limiting exposure to mycotoxins is the use of plasma. Plasma is an ionised gas that generates photons, positive and negative ions, and reactive oxygen and nitrogen species [110]. The ability of plasma to inactivate fungal growth and mycotoxin production has been well documented; nevertheless, mycotoxin degradability has also been investigated recently in some studies [111]. This method has significant limitations in its specific application. Plasma equipment is still in the stage of laboratory testing and standardisation [112].

An innovative way to remove mycotoxins from food is the use of nanozymes. Nanozymes are inorganic nanoparticles with properties similar to enzymes in redox reactions. They have been developed to remove pollutants, including AFB1 [113,114]. Nanozymes combine adsorption properties with filtration, adsorption and catalysis process [115,116,117]. A high yield (96%) and low impact on product quality were obtained in vegetable oils [117]. In order to introduce the practical application of nanozymes, further studies of this method are necessary.

Climate change remains a significant problem in reducing food contamination by mycotoxins. Global and local changes in temperature, humidity and CO_2_ levels in the atmosphere, along with extreme weather events such as floods and droughts are potential threats to both growers and food producers when it comes to mycotoxin prevention [118]. The rates of aflatoxin contamination in maize are already of concern, as are the increasing levels of mycotoxins produced by *Fusarium graminearum* in crops grown in various parts of Europe [119]. A similar problem affects winemakers; climate change may increase the vulnerability of grapes to fungal diseases, ultimately leading to increased mycotoxin contamination of their products [120]. 

In recent years, researchers have pointed out that mycotoxin levels may be underestimated as a result of the so-called “modified” or “masked” mycotoxins in food (e.g., zearalenone-14-glucoside (ZEA-14-Glc), ZEA-14-S). These toxins go undetected during routine analysis, which is usually utilized to detect parent toxins. The modified form of mycotoxins can be produced by fungi or plants as part of plant metabolic defenses by conjugating small polar molecules to the parent toxin during the growth period. Nevertheless, these substances can be hydrolyzed to precursor mycotoxins during human digestion. Toxicological data are sparse, but few studies to date have identified the potential health safety hazards of these toxins in animals. It has been shown that the modified forms of ZEA, ZEA-14-Glc and ZEA-16-Glc have a lower toxicity than the basic form of ZEA when adding a sugar moiety to the parent toxin; therefore, this metabolite has no affinity for oestrogen receptors [121]. Another form, ZEA-14-S, also showed no oestrogenic properties in studies on cell lines [122]. On the other hand, the hydroxylated forms of ZEA, namely, α-zearalenol (α-ZEA) and β-zearalenol (β-ZEA), show varied oestrogenic potential. α-ZEA has a potential up to 60 times higher than the basic form, while β-ZEA is lower [123]. Modified forms of deoxynivalenol from *Fusarium* spp. (as ZEA), i.e., deoxynivalenol-3-glucoside, 3-acetyl-deoxynivalenol and 15-acetyl-deoxynivalenol, have been thoroughly studied for their toxicity and hazards to human health [124].

It is important that future considerations should focus on detailed toxicological studies of the co-existence of basic and modified mycotoxins and setting limits for the presence of modified forms.

## 3. Conclusions

Mycotoxins are present in the environment, including food, and efforts should be made by the scientific community, including clinicians, to increase our knowledge of mycotoxin exposure and the related human health risks. Existing interactions between mycotoxins and existing food contaminants, i.e., pesticides, heavy metals or residues of veterinary drugs, remain a significant problem. In addition, the effect of mycotoxins with concurrent hormonal therapy should be taken into account in the safety assessment. We believe that a detailed prospective and epidemiological studies using linked databases are needed to support risk management strategies to reduce exposure to mycotoxins.

## Data Availability

The data presented in this study are available in this article.

## References

[1] UNEP, WHO (2012). State of the Science of Endocrine Disrupting Chemicals—2012.

[2] Kowalska K., Habrowska-Górczyńska D.E., Piastowska-Ciesielska A.W. (2016). Zearalenone as an Endocrine Disruptor in Humans. Environ. Toxicol. Pharmacol..

[3] Council for Agricultural Science and Technology (CAST) (2003). Mycotoxins, Risks in Plants, Animal and Human System.

[4] Gadzała-Kopciuch R., Cendrowski K., Cesarz A., Kiełbasa P., Buszewski B. (2011). Determination of Zearalenone and Its Metabolites in Endometrial Cancer by Coupled Separation Techniques. Anal. Bioanal. Chem..

[5] Mostrom M.S., Gupta R. (2012). Zearalenone. Veterinary Toxicology: Basic and Clinical Principles.

[6] Fink-Gremmels J., Malekinejad H. (2007). Clinical effects and biochemical mechanisms associated with exposure to the mycoestrogen zearalenone. Anim. Feed Sci. Technol..

[7] Zinedine A., Soriano J.M., Moltó J.C., Mañes J. (2007). Review on the toxicity, occurrence, metabolism, detoxification, regulations and intake of zearalenone: An oestrogenic mycotoxin. Food Chem. Toxicol..

[8] Ueberschär K.-H., Brezina U., Dänicke S. (2016). Zearalenone (ZEN) and ZEN metabolites in feed, urine and bile of sows: Analysis, determination of the metabolic profile and evaluation of the binding forms. Appl. Agric. For. Res..

[9] Wang Y., Zheng W., Bian X., Yuan Y., Gu J., Liu X., Liu Z., Bian J. (2014). Zearalenone Induces Apoptosis and Cytoprotective Autophagy in Primary Leydig Cells. Toxicol. Lett..

[10] Frizzell C., Ndossi D., Verhaegen S., Dahl E., Eriksen G., Sørlie M., Ropstad E., Muller M., Elliott C.T., Connolly L. (2011). Endocrine Disrupting Effects of Zearalenone, Alpha- and Beta-Zearalenol at the Level of Nuclear Receptor Binding and Steroidogenesis. Toxicol. Lett..

[11] Pfeiffer E., Kommer A., Dempe J.S., Hildebrand A.A., Metzler M. (2010). Absorption and Metabolism of the Mycotoxin Zearalenone and the Growth Promotor Zeranol in Caco-2 Cells in Vitro. Mol. Nutr. Food Res..

[12] So M.Y., Tian Z., Phoon Y.S., Sha S., Antoniou M.N., Zhang J., Wu R.S.S., Tan-Un K.C. (2014). Gene Expression Profile and Toxic Effects in Human Bronchial Epithelial Cells Exposed to Zearalenone. PLoS ONE.

[13] Molina-Molina J.-M., Real M., Jimenez-Diaz I., Belhassen H., Hedhili A., Torné P., Fernández M.F., Olea N. (2014). Assessment of Estrogenic and Anti-Androgenic Activities of the Mycotoxin Zearalenone and Its Metabolites Using In Vitro Receptor-Specific Bioassays. Food Chem. Toxicol..

[14] Groopman J.D., Egner P.A., Schulze K.J., Wu L.S.-F., Merrill R., Mehra S., Shamim A.A., Ali H., Shaikh S., Gernand A. (2014). Aflatoxin Exposure during the First 1000 Days of Life in Rural South Asia Assessed by Aflatoxin B1-Lysine Albumin Biomarkers. Food Chem. Toxicol..

[15] Sansen S., Yano J.K., Reynald R.L., Schoch G.A., Griffin K.J., Stout C.D., Johnson E.F. (2007). Adaptations for the Oxidation of Polycyclic Aromatic Hydrocarbons Exhibited by the Structure of Human P450 1A2. J. Biol. Chem..

[16] Jiang H., Wu J., Zhang F., Wen J., Jiang J., Deng Y. (2018). The Critical Role of Porcine Cytochrome P450 3A46 in the Bioactivation of Aflatoxin B1. Biochem. Pharmacol..

[17] Kolawole O., Meneely J.P., Meneely J.P., Petchkongkaew A., Elliott C. (2021). A review of myco-toxin biosynthetic pathways: Associated genes and their expressions under the influence of climatic factors. Fungal Biol. Rev..

[18] Martin J.F., van den Berg M.A., Ver Loren van Themaat E., Liras P. (2019). Sensing and transduction of nutritional and chemical signals in filamentous fungi: Impact on cell development and secondary metabolites bio-synthesis. Biotechnol. Adv..

[19] Loi M., Logrieco A.F., Pusztahelyi T., Leiter É., Hornok L., Pócsi I. (2023). Advanced myco-toxin control and decontamination techniques in view of an increased aflatoxin risk in Europe due to climate change. Front. Microbiol..

[20] Williams J.H., Phillips T.D., Jolly P.E., Stiles J.K., Jolly C.M., Aggarwal D. (2004). Human Aflatoxicosis in Developing Countries: A Review of Toxicology, Exposure, Potential Health Consequences, and Interventions. Am. J. Clin. Nutr..

[21] Gore A.C., Chappell V.A., Fenton S.E., Flaws J.A., Nadal A., Prins G.S., Toppari J., Zoeller R.T. (2015). EDC-2: The Endocrine Society’s Second Scientific Statement on Endocrine-Disrupting Chemicals. Endocr. Rev..

[22] Meeker J.D. (2012). Exposure to Environmental Endocrine Disruptors and Child Development. Arch. Pediatr. Adolesc. Med..

[23] Lucchese T.A., Grunow N., Ian Werner I., de Jesus A.L., Arbex A.K. (2017). Endocrine disruptors and fetal programming. OJEMD.

[24] Li L., Wu X., Guan H., Mao B., Wang H., Yuan X., Chu Y., Sun J., Ge R.-S. (2015). Zearalenone Inhibits Rat and Human 11β-Hydroxysteroid Dehydrogenase Type 2. BioMed Res. Int..

[25] Obremski K., Gonkowski S., Wojtacha P. (2015). Zearalenone-Induced Changes in the Lymphoid Tissue and Mucosal Nerve Fibers in the Porcine Ileum. Pol. J. Vet. Sci..

[26] World Health Organization (WHO) Endocrine Disorders and Children, Children’s Health and the Environment. http://www.portal.pmnch.org/ceh/capacity/endocrine.pdf.

[27] Yum T., Lee S., Kim Y. (2013). Association between Precocious Puberty and Some Endocrine Disruptors in Human Plasma. J. Environ. Sci. Health A.

[28] Massart F., Meucci V., Saggese G., Soldani G. (2008). High Growth Rate of Girls with Precocious Puberty Exposed to Estrogenic Mycotoxins. J. Pediatr..

[29] Massart F., Saggese G. (2010). Oestrogenic Mycotoxin Exposures and Precocious Pubertal Development. Int. J. Androl..

[30] Bandera E.V., Chandran U., Buckley B., Lin Y., Isukapalli S., Marshall I., King M., Zarbl H. (2011). Urinary mycoestrogens, body size and breast development in New Jersey girls. Sci. Total Environ..

[31] Rivera-Núñez Z., Barrett E.S., Szamreta E.A., Shapses S.A., Qin B., Lin Y., Zarbl H., Buckley B., Bandera E.V. (2019). Urinary Mycoestrogens and Age and Height at Menarche in New Jersey Girls. Environ. Health.

[32] Asci A., Durmaz E., Erkekoglu P., Pasli D., Bircan I., Kocer-Gumusel B. (2014). Urinary Zearalenone Levels in Girls with Premature Thelarche and Idiopathic Central Precocious Puberty. Minerva Pediatr..

[33] Szuets P., Mesterházy Á., Falkay G., Bartók T. (1997). Early Telarche Symptoms in Children and Their Relations to Zearalenon Contamination in Foodstuffs. Cereal Res. Commun..

[34] Zheng W., Feng N., Wang Y., Noll L., Xu S., Liu X., Lu N., Zou H., Gu J., Yuan Y. (2019). Effects of Zearalenone and Its Derivatives on the Synthesis and Secretion of Mammalian Sex Steroid Hormones: A Review. Food Chem. Toxicol Int. J. Publ. Br. Ind. Biol. Res. Assoc..

[35] Zwierzchowski W., Przybyłowicz M., Obremski K., Zielonka L., Skorska-Wyszyńska E., Gajecka M., Polak M., Jakimiuk E., Jana B., Rybarczyk L. (2005). Level of Zearalenone in Blood Serum and Lesions in Ovarian Follicles of Sexually Immature Gilts in the Course of Zearalenone Micotoxicosis. Pol. J. Vet. Sci..

[36] Jakimiuk E., Rybarczyk L., Zwierzchowski W., Obremski K., Gajęcka M., Zielonka Ł., Gajęcki M. (2010). Effect of experimental long-term exposure to low-dose zearalenone mycotoxicosis on selected morphometric parameters of the reproductive tract in sexually-immature gilts. Bull. Vet. Inst. Pulawy.

[37] Caserta D., Mantovani A., Marci R., Fazi A., Ciardo F., La Rocca C., Maranghi F., Moscarini M. (2011). Environment and Women’s Reproductive Health. Hum. Reprod. Update.

[38] Balló A., Busznyákné Székvári K., Czétány P., Márk L., Török A., Szántó Á., Máté G. (2023). Estrogenic and Non-Estrogenic Disruptor Effect of Zearalenone on Male Reproduction: A Review. Int. J. Mol. Sci..

[39] Ibeh I.N., Uraih N., Ogonor J.I. (1991). Dietary exposure to aflatoxin in Benin City, Nigeria: A possible public health concern. Int. J. Food Microbiol..

[40] Uriah N., Ibeh I.N., Oluwafemi F. (2001). A Study on the Impact of Aflatoxin on Human Reproduction. AJRH.

[41] Kinkade C.W., Rivera-Núñez Z., Gorcyzca L., Aleksunes L.M., Barrett E.S. (2021). Impact of Fusarium-Derived Mycoestrogens on Female Reproduction: A Systematic Review. Toxins.

[42] Prouillac C., Videmann B., Mazallon M., Lecoeur S. (2009). Induction of Cells Differentiation and ABC Transporters Expression by a Myco-Estrogen, Zearalenone, in Human Choriocarcinoma Cell Line (BeWo). Toxicology.

[43] Prouillac C., Koraichi F., Videmann B., Mazallon M., Rodriguez F., Baltas M., Lecoeur S. (2012). In Vitro Toxicological Effects of Estrogenic Mycotoxins on Human Placental Cells: Structure Activity Relationships. Toxicol. Appl. Pharmacol..

[44] Partanen H.A., El-Nezami H.S., Leppänen J.M., Myllynen P.K., Woodhouse H.J., Vähäkangas K.H. (2010). Aflatoxin B1 Transfer and Metabolism in Human Placenta. Toxicol. Sci..

[45] Warth B., Preindl K., Manser P., Wick P., Marko D., Buerki-Thurnherr T. (2019). Transfer and Metabolism of the Xenoestrogen Zearalenone in Human Perfused Placenta. Environ. Health Perspect..

[46] Maxwell S.M., Familusi J.B., Sodeinde O., Chan M.C., Hendrickse R.G. (1994). Detection of Naphthols and Aflatoxins in Nigerian Cord Blood. Ann. Trop. Paediatr..

[47] Abulu E.O., Uriah N., Aigbefo H.S., Oboh P.A., Agbonlahor D.E. (1998). Preliminary Investigation on Aflatoxin in Cord Blood of Jaundiced Neonates. West. Afr. J. Med..

[48] Abdulrazzaq Y.M., Osman N., Ibrahim A. (2002). Fetal Exposure to Aflatoxins in the United Arab Emirates. Ann. Trop. Paediatr..

[49] Abdulrazzaq Y.M., Osman N., Yousif Z.M., Trad O. (2004). Morbidity in Neonates of Mothers Who Have Ingested Aflatoxins. Ann. Trop. Paediatr..

[50] Turner P.C., Collinson A.C., Cheung Y.B., Gong Y., Hall A.J., Prentice A.M., Wild C.P. (2007). Aflatoxin Exposure in Utero Causes Growth Faltering in Gambian Infants. Int. J. Epidemiol..

[51] Shuaib F.M.B., Person S.D., Funkhouser E., Yatich N.J., Stiles J.K., Ellis W.O., Jiang Y., Ehiri J.E., Williams J.H., Jolly P.E. (2010). Association between Anemia and Aflatoxin B1 Biomarker Levels among Pregnant Women in Kumasi, Ghana. Am. J. Trop. Med. Hyg..

[52] Sodeinde O., Chan M.C., Maxwell S.M., Familusi J.B., Hendrickse R.G. (1995). Neonatal Jaundice, Aflatoxins and Naphthols: Report of a Study in Ibadan, Nigeria. Ann. Trop. Paediatr..

[53] Ahmed H., Hendrickse R.G., Maxwell S.M., Yakubu A.M. (1995). Neonatal Jaundice with Reference to Aflatoxins: An Aetiological Study in Zaria, Northern Nigeria. Ann. Trop. Paediatr..

[54] Shuaib F.M.B., Jolly P.E., Ehiri J.E., Yatich N., Jiang Y., Funkhouser E., Person S.D., Wilson C., Ellis W.O., Wang J.-S. (2010). Association between Birth Outcomes and Aflatoxin B_1_Biomarker Blood Levels in Pregnant Women in Kumasi, Ghana. Trop. Med. Int. Health.

[55] Kristensen P., Irgens L.M., Andersen A., Bye A.S., Sundheim L. (1997). Gestational Age, Birth Weight, and Perinatal Death among Births to Norwegian Farmers, 1967-1991. Am. J. Epidemiol..

[56] Kristensen P., Andersen A., Irgens L.M. (2000). Hormone-Dependent Cancer and Adverse Reproductive Outcomes in Farmers’ Families—Effects of Climatic Conditions Favoring Fungal Growth in Grain. Scand. J. Work Environ. Health.

[57] Nordby K.-C., Irgens L.M., Kristensen P. (2006). Immunological Exposures in Norwegian Agriculture and Pre-Eclampsia. Paediatr. Perinat. Epidemiol..

[58] Carlos R.L.J., Leticia I.G., Efrain F.S.E., Miguel R.A. (2015). Aflatoxigenic Feeding and Its Possible Implications after Pregnancy. Biomed. Pharmacol. J..

[59] Omotayo O.P., Omotayo A.O., Mwanza M., Babalola O.O. (2019). Prevalence of Mycotoxins and Their Consequences on Human Health. Toxicol. Res..

[60] Missmer S.A., Suarez L., Felkner M., Wang E., Merrill A.H., Rothman K.J., Hendricks K.A. (2006). Exposure to Fumonisins and the Occurrence of Neural Tube Defects along the Texas–Mexico Border. Environ. Health Perspect..

[61] Smith L.E., Prendergast A.J., Turner P.C., Humphrey J.H., Stoltzfus R.J. (2017). Aflatoxin Exposure during Pregnancy, Maternal Anemia, and Adverse Birth Outcomes. Am. J. Trop. Med. Hyg..

[62] Hagberg H., Mallard C., Jacobsson B. (2005). Role of Cytokines in Preterm Labour and Brain Injury. BJOG Int. J. Obstet. Gynaecol..

[63] Wang Y., Li L., Wang C.C., Leung L.K. (2013). Effect of Zeranol on Expression of Apoptotic and Cell Cycle Proteins in Murine Placentae. Toxicology.

[64] Andrews-Trevino J.Y., Webb P., Shively G., Rogers B.L., Baral K., Davis D., Paudel K., Pokharel A., Shrestha R., Wang J.-S. (2019). Relatively Low Maternal Aflatoxin Exposure Is Associated with Small-For-Gestational-Age but Not with Other Birth Outcomes in a Prospective Birth Cohort Study of Nepalese Infants. J. Nutr..

[65] Andretta I., Kipper M., Lehnen C.R., Lovatto P.A. (2012). Meta-Analysis of the Relationship of Mycotoxins with Biochemical and Hematological Parameters in Broilers. Poult. Sci..

[66] Yousef M.I., Salem M.H., Kamel K.I., Hassan G.A., El-Nouty F.D. (2003). Influence of Ascorbic Acid Supplementation on the Haematological and Clinical Biochemistry Parameters of Male Rabbits Exposed to Aflatoxin B_1_. J. Environ. Sci. Health Part B.

[67] Eisa A., Metwally A. (2011). Effect of Glucomannan on Haematological, Coagulation and Biochemical Parameters in Male Rabbits Fed Aflatoxin-Contaminated Ration. World Mycotoxin J..

[68] Moodley D., Moodley L., Reddy M.F., Dutton A.A., Chuturgoon J. (2001). Fumonisin B 1: An Aetiological Role in Pre-Eclampsia. J. Obstet. Gynaecol..

[69] Pazaiti A., Kontos M., Fentiman I.S. (2011). ZEN and the Art of Breast Health Maintenance. Int. J. Clin. Pract..

[70] Fernandez S.V., Russo J. (2009). Estrogen and Xenoestrogens in Breast Cancer. Toxicol. Pathol..

[71] Fucic A., Gamulin M., Ferencic Z., Katic J., Krayer von Krauss M., Bartonova A., Merlo D.F. (2012). Environmental Exposure to Xenoestrogens and Oestrogen Related Cancers: Reproductive System, Breast, Lung, Kidney, Pancreas, and Brain. Environ. Health.

[72] Rogowska A., Pomastowski P., Sagandykova G., Buszewski B. (2019). Zearalenone and Its Metabolites: Effect on Human Health, Metabolism and Neutralisation Methods. Toxicon.

[73] Kuciel-Lisieska G., Obremski K., Stelmachów J., Gajecka M., Zielonka Ł., Jakimiuk E., Gajecki M. (2008). Presence of zearalenone in blood plasma in women with neoplastic lesions in the mammary gland. Bul. Vet. Inst. Pulawy.

[74] Belhassen H., Jiménez-Díaz I., Arrebola J.P., Ghali R., Ghorbel H., Olea N., Hedili A. (2015). Zearalenone and Its Metabolites in Urine and Breast Cancer Risk: A Case-Control Study in Tunisia. Chemosphere.

[75] Pillay D., Chuturgoon A.A., Nevines E., Manickum T., Deppe W., Dutton M.F. (2002). The Quantitative Analysis of Zearalenone and Its Derivatives in Plasma of Patients with Breast and Cervical Cancer. Clin. Chem. Lab. Med..

[76] Wan M.L.Y., Co V.A., El-Nezami H. (2022). Endocrine Disrupting Chemicals and Breast Cancer: A Systematic Review of Epidemiological Studies. Crit. Rev. Food Sci. Nutr..

[77] Pajewska M., Łojko M., Cendrowski K., Sawicki W., Kowalkowski T., Buszewski B., Gadzała-Kopciuch R. (2018). The Determination of Zearalenone and Its Major Metabolites in Endometrial Cancer Tissues. Anal. Bioanal. Chem..

[78] Marchese S., Polo A., Ariano A., Velotto S., Costantini S., Severino L. (2018). Aflatoxin B1 and M1: Biological Properties and Their Involvement in Cancer Development. Toxins.

[79] Caserta D., De Marco M.P., Besharat A.R., Costanzi F. (2022). Endocrine Disruptors and Endometrial Cancer: Molecular Mechanisms of Action and Clinical Implications, a Systematic Review. Int. J. Mol. Sci..

[80] Eze U.A., Okonofua F.E. (2015). High Prevalence of Male Infertility in Africa: Are Mycotoxins to Blame?. Afr. J. Reprod. Health.

[81] Mally A., Solfrizzo M., Degen G.H. (2016). Biomonitoring of the mycotoxin Zearalenone: Current state-of-the art and application to human exposure assessment. Arch. Toxicol..

[82] Al-Jaal B.A., Jaganjac M., Barcaru A., Horvatovich P., Latiff A. (2019). Aflatoxin, fumonisin, ochratoxin, zearalenone and deoxynivalenol biomarkers in human biological fluids: A systematic literature review, 2001–2018. Food Chem. Toxicol..

[83] Fleck S.C., Churchwell M.I., Doerge D.R., Teeguarden J.G. (2016). Urine and serum biomonitoring of exposure to environmental estrogens II: Soy isoflavones and zearalenone in pregnant women. Food Chem. Toxicol..

[84] Mirocha C.J., Pathre S.V., Robison T.S. (1981). Comparative Metabolism of Zearalenone and Transmission into Bovine Milk. Food Cosmet. Toxicol..

[85] Warth B., Sulyok M., Berthiller F., Schuhmacher R., Krska R. (2013). New Insights into the Human Metabolism of the Fusarium Mycotoxins Deoxynivalenol and Zearalenone. Toxicol. Lett..

[86] Ferrigo D., Raiola A., Causin R. (2016). Fusarium Toxins in Cereals: Occurrence, Legislation, Factors Promoting the Appearance and Their Management. Molecules.

[87] McKenzie K.S., Sarr A.B., Mayura K., Bailey R.H., Miller D.R., Rogers T.D., Norred W.P., Voss K.A., Plattner R.D., Kubena L.F. (1997). Oxidative Degradation and Detoxification of Mycotoxins Using a Novel Source of Ozone. Food Chem. Toxicol..

[88] Dalié D.K.D., Deschamps A.M., Richard-Forget F. (2010). Lactic acid bacteria—Potential for control of mould growth and mycotoxins: A review. Food Control.

[89] Vinderola G., Ritieni A. (2014). Role of probiotics against mycotoxins and their deleterious effects. J. Food Res..

[90] Abdolmaleki K., Javanmardi F., Gavahian M., Phimolsiripol Y., Ruksiriwanich W., Mir S.A. (2022). Emerging technologies in combination with probiotics for aflatoxins removal: An updated review. Int. J. Food Sci..

[91] Sangsila A., Faucet-Marquis V., Pfohl-Leszkowicz A., Itsaranuwat P. (2016). Detoxification of zearalenone by *Lactobacillus pentosus* strains. Food Control.

[92] Harkai P., Szabó I., Cserháti M., Krifaton C., Risa A., Radó J., Balázs A., Berta K., Kriszt B. (2016). Biodegradation of aflatoxin-B1, and zearalenone by *Streptomyces* sp. collection. Int. Biodeterior. Biodegrad..

[93] Wang Y., Zhao C., Zhang D., Zhao M., Peng M., Guo P., Cui Z. (2020). Microbial degradation of zearalenone by a novel microbial consortium, NZDC-6, and its application on contaminated corncob by semisolid fermentation. J. Agric. Food Chem..

[94] Wang Y., Zhao C., Zhang D., Zhao M., Zheng D., Lyu Y., Cheng W., Guo P., Cui Z. (2017). Effective degradation of aflatoxin B1, using a novel thermophilic microbial consortium TADC7. Bioresour. Technol..

[95] Wang Y., Zhao C., Zhang D., Zhao M., Zheng D., Peng M., Cheng W., Guo P., Cui Z. (2018). Simultaneous degradation of aflatoxin B1, and zearalenone by a microbial consortium. Toxicon.

[96] Yu Y., Wu H., Tang Y., Qiu L. (2012). Cloning, expression of a peroxiredoxin gene from *Acinetobacter* sp. SM04, and characterization of its recombinant protein for zearalenone detoxification. Microbiol. Res..

[97] Rawal S., Yip S.S.M., Coulombe R.A. (2010). Cloning, expression and functional characterization of cytochrome P450, 3A37, from turkey liver with high aflatoxin B1, epoxidation activity. Chem. Res. Toxicol..

[98] Ren X., Branà M.T., Haidukowski M., Gallo A., Zhang Q., Logrieco A.F. (2022). Potential of *Trichoderma* spp. for biocontrol of aflatoxin-producing *Aspergillus flavus*. Toxins.

[99] Gamal M., Abou Zaid M., Abou Mourad I.K., Abd El Kareem H., Gomaa O.M. (2022). *Trichoderma viride* bioactive peptaibol induces apoptosis in *Aspergillus niger* in-fecting tilapia in fish farms. Aquaculture.

[100] Woo S.L., Ruocco M., Vinale F., Nigro M., Marra R., Lombardi N. (2014). Trichoderma-based products and their widespread use in agriculture. Open Mycol. J..

[101] Sun S., Zhao R., Xie Y., Liu Y. (2019). Photocatalytic degradation of aflatoxin B1 by activated carbon supported TiO2 catalyst. Food Control.

[102] Faraji H., Yazdi F.T., Razmi N. (2022). The influence of ultraviolet radiation on aflatoxin producing *Aspergillus* species’ isolated from Iranian rice. Toxicol. Rep..

[103] Calado T., Abrunhosa L., Cabo Verde S., Alté L., Venâncio A., Fernández-Cruz M.L. (2020). Effect of Gamma-Radiation on Zearalenone-Degradation, Cytotoxicity and Estrogenicity. Foods.

[104] Kalagatur N.K., Kamasani J.R., Mudili V. (2018). Assessment of detoxification efficacy of irradiation on zearalenone mycotoxin in various fruit juices by response surface methodology and elucidation of its in-vitro toxicity. Front. Microbiol..

[105] Shen M.H., Singh R.K. (2022). Effective UV wavelength range for increasing aflatoxins reduction and decreasing oil deterioration in contaminated peanuts. Food Res. Int..

[106] Horky P., Skalickova S., Baholet D., Skladanka J. (2018). Nanoparticles as a Solution for Eliminating the Risk of Mycotoxins. Nanomaterials.

[107] Duishemambet Kyzy A., Kocyigit Y., Ardag A.H. (2022). Aflatoxin B1 bioremoval by fungal cells immobilised on magnetic nanoparticles. J. Environ. Anal. Chem..

[108] Lu D., Tang S., Li Y., Cong Z., Zhang X., Wu S. (2021). Magnetic-propelled Janus yeast cell robots functionalized with metal-organic frameworks for mycotoxin decontamination. Micromachines.

[109] Malhotra N., Lee J.S., Liman R.A.D., Ruallo J.M.S., Villaflores O.B., Ger T.R. (2020). Potential toxicity of iron oxide magnetic nanoparticles: A review. Molecules.

[110] Mandal R., Singh A., and Pratap S.A. (2018). Recent developments in cold plasma decontamination technology in the food industry. Trends Food Sci. Technol..

[111] Hojnik N., Modic M., Walsh J.L., Žigon D., Javornik U., Plavec J., Zegura B., Filipic M., Cvelbar U. (2021). Unravelling the pathways of air plasma induced aflatoxin B1 degradation and detoxification. J. Hazard. Mater..

[112] Wu Y., Cheng J.H., Sun D.W. (2021). Blocking and degradation of aflatoxins by cold plasma treatments: Applications and mechanisms. Trends Food Sci. Technol..

[113] Zhang X., Li G., Wu D., Liu J., Wu Y. (2020). Recent advances on emerging nanomaterials for controlling the mycotoxin contamination: From detection to elimination. Food Front..

[114] Guo Y., Zhao L., Ma Q., Ji C. (2021). Novel strategies for degradation of aflatoxins in food and feed: A review. Food Res. Int..

[115] Ma F., Cai X., Mao J., Yu L., Li P. (2021). Adsorptive removal of aflatoxin B1 from vegetable oils via novel adsorbents derived from a metal-organic framework. J. Hazard. Mater..

[116] Pérez-Gómez E.O., García-Rosales G., Longoria-Gándara L.C., Gómez-Vilchis J.C. (2022). Obtention of biochar-Ca nanoparticles using citrus tangerina: A morphological, surface and study remotion of aflatoxin AFB1. J. Hazard. Mater..

[117] Wei J., Wu X., Wu C., Hou F., Wu L., Huang H. (2022). Metal-organic frameworks with peroxidase-like activity for efficient removal of aflatoxin B1. Food Chem..

[118] Zingales V., Taroncher M., Martino P.A., Ruiz M.J., Caloni F. (2022). Climate Change and Effects on Molds and Mycotoxins. Toxins..

[119] Battilani P., Toscano P., Van der Fels-Klerx H.J., Moretti A., Camardo Leggieri M., Brera C., Rortais A., Goumperis T., Robinson T. (2016). Aflatoxin B1 contamination in maize in Europe increases due to climate change. Sci. Rep..

[120] Garcia-Cela E., Crespo-Sempere A., Ramos A.J., Sanchis V., Marin S. (2014). Ecophysiological characterization of *Aspergillus carbonarius*, *Aspergillus tubingensis* and *Aspergillus niger* isolated from grapes in Spanish vineyards. Int. J. Food Microbiol..

[121] Poppenberger B., Berthiller F., Bachmann H., Lucyshyn D., Peterbauer C., Mitterbauer R., Schuhmacher R., Krska R., Glössl J., Adam G. (2006). Heterologous expression of Arabidopsis UDP-glucosyltransferases in *Saccharomyces cerevisiae* for production of zearalenone-4-O-glucoside. Appl. Environ. Microbiol..

[122] Gratz S.W. (2017). Do Plant-Bound Masked Mycotoxins Contribute to Toxicity?. Toxins.

[123] Knutsen H.K., Barregård L., Bignami M., Brüschweiler B., Ceccatelli S., Cottrill B., Dinovi M., Edler L., Grasl-Kraupp B., Hogstrand C. (2018). Appropriateness to set a group health-based guidance value for fumonisins and their modified forms. EFSA J..

[124] Sun Y., Jiang J., Mu P., Lin R., Wen J., Deng Y. (2022). Toxicokinetics and metabolism of deoxynivalenol in animals and humans. Arch. Toxicol..

